# Longitudinal Plasma Metabolomics by GC–MS and LC–MS During Total Parenteral Nutrition After Gastrointestinal Surgery

**DOI:** 10.3390/metabo16030199

**Published:** 2026-03-16

**Authors:** Duygu Konuklu, Cemil Can Eylem, İpek Baysal, Busenur Kırımtay, Emirhan Nemutlu, Timuçin Erol, Şermin Ataç, İncilay Süslü

**Affiliations:** 1 Department of Analytical Chemistry, Faculty of Pharmacy, Hacettepe University, 06100 Ankara, Turkey; duygukonuklu@gmail.com (D.K.); cemilcaneylem@hacettepe.edu.tr (C.C.E.); enemutlu@hacettepe.edu.tr (E.N.); 2 Department of Biochemistry, Faculty of Pharmacy, Hacettepe University, 06100 Ankara, Turkey; ipekbaysal@hacettepe.edu.tr; 3 Department of General Surgery, Faculty of Medicine, Hacettepe University, 06100 Ankara, Turkey; busenurkirimtay@hacettepe.edu.tr (B.K.); timucinerol@hacettepe.edu.tr (T.E.); 4 Nutritional Support Department, Hacettepe University Hospital, 06100 Ankara, Turkey; srmnatac@gmail.com

**Keywords:** time-resolved metabolomics, untargeted metabolomics, total parenteral nutrition, interventional metabolomics, GC-MS, LC-MS

## Abstract

Background: Total parenteral nutrition (TPN) is widely used after major gastrointestinal surgery; however, its early systemic metabolic effects and temporal adaptation patterns remain incompletely characterized. This study applied a longitudinal plasma metabolomics approach to investigate time-dependent metabolic changes during early TPN administration. Methods: Plasma samples were collected from patients undergoing gastrointestinal surgery before TPN initiation (baseline, T0) and at 24 h (T1), 48 h (T2), and 72 h (T3). Untargeted metabolomic profiling was performed using complementary gas chromatography–mass spectrometry (GC–MS) and liquid chromatography–mass spectrometry (LC–MS) platforms. In total, 111 metabolites were detected. Analysis of variance (ANOVA) with baseline (T0) as the reference identified time-point–specific metabolic alterations during TPN administration. Results: At 24 h (T1), nominally significant increases were observed in glycine, tryptophan, isoleucine, and methionine, accompanied by decreases in sarcosine and oxalic acid. At 48 h (T2), elevated levels of glycine, isoleucine, valine, and phenylalanine persisted, while sarcosine, oxalic acid, and myo-inositol remained decreased. By 72 h (T3), sustained increases in glycine, isoleucine, valine, phenylalanine, proline, alanine, and tryptophan were accompanied by reduced levels of sarcosine, oxalic acid, and glucopyranose, reflecting coordinated alterations across multiple metabolite classes. Conclusions: Overall, the results demonstrated a distinct longitudinal metabolomic pattern characterized by increases in circulating amino acids and time-dependent changes in carbohydrate- and lipid-related metabolites within the first 72 h of TPN. This exploratory, time-resolved metabolomic study in 37 patients highlights the utility of untargeted metabolomics for characterizing early metabolic adaptation to parenteral nutrition and supporting postoperative metabolic monitoring.

## 1. Introduction

Total parenteral nutrition (TPN) is an intravenous method of providing complete nutrition, including macro- and micronutrients such as glucose, amino acids, lipids, electrolytes, vitamins, and trace elements, directly into the bloodstream. It is used for patients who cannot obtain adequate nutrition through oral or enteral feeding due to gastrointestinal dysfunction or contraindications to enteral nutrition [1]. TPN formulas usually combine amino acid solutions with dextrose and lipid emulsions to fulfill energy and substrate needs in these cases [1]. TPN is often employed after major gastrointestinal surgery when enteral feeding is not possible, is contraindicated, or does not meet metabolic demands, especially when a complete gastrointestinal tract bypass is necessary due to prolonged postoperative ileus or bowel issues [2]. During the early postoperative phase, patients undergoing major surgery typically experience a stress-related metabolic response marked by hypermetabolism, increased proteolysis, heightened hepatic gluconeogenesis, disrupted lipid metabolism, insulin resistance, and a negative nitrogen balance [3,4,5]. These changes are part of a coordinated neuroendocrine and inflammatory response and, if prolonged or poorly managed, can hinder recovery and lead to worse clinical outcomes, highlighting the importance of prompt and suitable nutritional support [3,4,5]. Clinical studies consistently demonstrate that excessive postoperative catabolism and skeletal muscle loss are linked to negative clinical outcomes, such as higher complication rates, longer hospital stays, and slower functional recovery, especially in high-risk surgical and critically ill patients [6,7].

Early initiation of TPN seeks to reduce this catabolic response by supplying external energy sources, amino acids, and lipids during gastrointestinal dysfunctions [1,5]. Guidelines from international clinical nutrition authorities, including the European Society for Clinical Nutrition and Metabolism (ESPEN), highlight the importance of providing prompt nutritional support for certain surgical patients. This support aims to improve nitrogen balance, decrease muscle protein breakdown, and enhance metabolic stability in the early postoperative period [2,3]. Despite following these guidelines and TPN’s common clinical application, the overall metabolic effects of early TPN, especially how they change over the first postoperative days, are not yet fully understood. While the metabolic response to surgical stress has been extensively described, considerably less is known about how systemic metabolism dynamically adapts during the early phase of postoperative TPN. In particular, longitudinal, time-resolved metabolomic data capturing the first 72 h of TPN administration remain scarce.

Most clinical studies of postoperative TPN rely on traditional biochemical markers (e.g., blood glucose, lactate, triglycerides, and nitrogen balance), which provide limited insight into how amino acid, carbohydrate, and lipid pathways change over time during early TPN treatment [8,9].

Metabolomics provides a comprehensive analytical approach that addresses this gap by profiling a wide array of low-molecular-weight metabolites and offering a broader view of metabolic states than traditional targeted assays, enabling detection of changes in amino acids, glycolytic intermediates, lipid molecules, and organic acids [8,10,11]. Recently, metabolomics has become more widely used in critical illness and surgical research to study metabolic stress responses, nutritional strategies, and disease severity [8].

Many metabolomics studies in postoperative or critically ill patients are limited by targeted analytical strategies or single time-point designs [12,13]. Although targeted approaches provide high analytical specificity, they are restricted to predefined metabolite panels and may miss broader, pathway-level metabolic changes induced by complex interventions such as total parenteral nutrition [14]. In contrast, untargeted metabolomics enables comprehensive, time-resolved assessment of systemic metabolic alterations, making it particularly well suited for studying complex clinical interventions [11,15].

Moreover, combining complementary analytical platforms is increasingly recognized as important for maximizing metabolite coverage across chemically diverse classes [10,12]. In particular, longitudinal profiling starting from a true pre-TPN postoperative baseline and extending across the first days of parenteral nutrition has been insufficiently characterized. Studies applying repeated sampling within the same patients and integrating complementary analytical platforms are therefore needed to better describe early systemic metabolic adaptation following initiation of TPN after gastrointestinal surgery.

In this study, we performed a longitudinal, untargeted plasma metabolomics strategy, complemented by GC–MS and LC–MS analyses, to characterize early metabolic changes during the first 72 h of TPN in patients undergoing gastrointestinal surgery. Plasma samples were obtained before TPN initiation (baseline, T0) and at 24 h (T1), 48 h (T2), and 72 h (T3) after surgery, allowing direct assessment of how systemic metabolic profiles evolve in response to parenteral nutrient support. Using combined multivariate, univariate, and time-resolved statistical analyses, we aimed to identify metabolite profile changes that are consistently altered during early TPN. This approach enables the detection of coordinated metabolic responses that are not captured by routine clinical markers, offering a detailed view of the systemic metabolic adjustments associated with postoperative parenteral nutrition.

## 2. Materials and Methods

### 2.1. Study Design and Patient Population

Plasma samples were collected following approval by the Non-Interventional Clinical Research Ethics Committee of Hacettepe University (GO 21/946). Adult patients (≥18 years) undergoing major gastrointestinal surgery in the Department of General Surgery at Hacettepe University Hospital and receiving postoperative TPN as part of standard clinical care were enrolled in this observational study, with no additional procedures beyond routine postoperative management. Patients receiving TPN in the absence of surgical intervention or those younger than 18 years of age were excluded. A total of 37 eligible patients were included in the metabolomic analyses, and written informed consent was obtained from all participants prior to enrollment. Patient demographics and clinical characteristics are summarized in Table 1. Primary surgical procedures were defined as the index gastrointestinal surgeries leading to postoperative total parenteral nutrition administration and were extracted from operative records. Clinical severity scores such as APACHE II or SOFA were not routinely collected in this surgical cohort.

Because baseline (T0) samples were obtained in the early postoperative period prior to initiation of TPN, baseline metabolomic profiles reflect postoperative metabolic status rather than a pre-surgical steady state. Accordingly, longitudinal comparisons in this study were designed to evaluate early postoperative metabolic trajectories in the context of ongoing recovery and parenteral nutritional support.

Baseline clinical covariates relevant to metabolic and inflammatory status, including C-reactive protein (CRP), liver enzymes (AST, ALT, GGT, LDH), renal function parameters (creatinine, estimated GFR), thyroid-stimulating hormone (TSH), coagulation indices (APTT, INR), and HbA1c, as well as major comorbidities and type of index surgery, were extracted from electronic medical records and summarized to provide clinical context for metabolomic analyses (Supplementary File S1, Table S1).

### 2.2. TPN Administration Protocol

TPN was initiated within 24 h after surgery in accordance with international clinical nutrition guidelines issued by the ESPEN and the American Society for Parenteral and Enteral Nutrition (ASPEN). The composition of the TPN solutions was individualized on a daily basis according to each patient’s clinical condition, nutritional requirements, and biochemical parameters. Typical formulations of TPN included glucose, amino acids, lipid emulsions, electrolytes, vitamins, and trace elements. All TPN solutions were administered via a central venous catheter using volumetric infusion pumps under aseptic conditions. Energy and macronutrient targets were calculated based on actual body weight, estimated stress factors, and metabolic demands, and were reassessed throughout the treatment period as clinically indicated. Because postoperative nutritional management is highly individualized, a single standardized TPN formulation was not used for all patients.

Macronutrient delivery (amino acids, dextrose, and lipids) was calculated as grams per kilogram per day (g/kg/day). For each patient, calculations were based on the recorded daily TPN administration data, including either total TPN volume or individual component volumes (amino acid, dextrose, and lipid), together with the quantitative composition of the commercial multi-chamber formulations used. Lipid emulsions were not standardized across the cohort; both olive oil/soybean oil-based and soybean oil-based lipid emulsions were used according to institutional practice.

### 2.3. Sample Collection and Preparation

Venous blood samples were collected at four predefined time points: prior to TPN initiation (baseline, T0), and at 24 h (T1), 48 h (T2), and 72 h (T3) following initiation of TPN. Blood samples were collected in EDTA-containing tubes and centrifuged at 3000 rpm for 10 min at room temperature. The plasma fraction was then aliquoted and temporarily stored at −20 °C immediately after centrifugation and subsequently transferred to −80 °C for long-term storage prior to metabolite extraction. The duration of −20 °C storage was minimized and standardized across all samples to avoid differential metabolite degradation. An aliquot of 200 µL plasma was transferred into an Eppendorf tube, and metabolites were extracted by protein precipitation using 800 µL of methanol:water (9:1, *v*/*v*). Samples were vortex-mixed for 30 s and centrifuged at 15,000 rpm at 4 °C for 10 min. The supernatant (400 µL) was separately collected for GC–MS and LC–MS analyses, evaporated to dryness, and stored at −80 °C until analysis. Quality control (QC) samples were prepared by pooling equal volumes of metabolite extracts from all study samples.

### 2.4. Metabolomic Analysis

#### 2.4.1. GC-MS Based Metabolomic Analysis

A total of 20 μL of methoxyamine hydrochloride (20 mg/mL in pyridine) was added to the dried samples, and the samples were incubated at 30 °C for 90 min in a dry block shaker to allow methoximation. After cooling to room temperature, 50 μL of N-methyl-N-(trimethylsilyl) trifluoroacetamide + 1% trimethylchlorosilane (MSTFA + 1% TMCS) was added, followed by incubation at 37 °C for 30 min to complete silylation. The derivatized samples were transferred into silanized GC–MS vials and analyzed using a GC–MS system (Shimadzu, Kyoto, Japan) equipped with a DB-5MS capillary column (30 m + 10 m × 0.25 mm i.d., 0.25 μm film thickness), as described in our previous study [16]. QC samples were injected periodically (every sixth sample) throughout the analytical sequence to monitor instrument stability, signal drift, and analytical reproducibility.

#### 2.4.2. LC-MS Based Metabolomic Analysis

The chromatographic separation was achieved on a reversed-phase C18 column (2.1 × 100 mm, 2.7 μm particle size) maintained at 30 °C. The mobile phases consisted of (A) water containing 0.1% formic acid and (B) acetonitrile containing 0.1% formic acid, and analytes were eluted using a gradient elution program at a flow rate of 0.3 mL/min over a total run time of 25 min. The injection volume was set to 10 μL. QC samples were injected periodically every sixth sample throughout the analytical sequence. qTOF-MS analysis parameters were as described in our previous study [17]. Briefly, mass spectrometric detection was performed in both positive and negative electrospray ionization (ESI) modes. Full-scan MS data were acquired at 100 and 1700 *m*/*z* (MS1) and 50–1700 *m*/*z* (MS2). The data-dependent MS/MS spectra were obtained from pooled quality control (QC) samples at 10, 20, and 40 eV collision energies to support metabolite annotation.

#### 2.4.3. Data Processing and Statistical Analysis

Complex chromatograms obtained from GC–MS and LC–MS-based untargeted metabolomics analyses were processed using MS-DIAL software (version 4.92). Peak detection, deconvolution, retention time alignment, and data matrix generation were performed separately for GC–MS and LC–MS raw data.

For GC–MS data, peak identification was performed by matching mass spectra and retention indices against the Fiehn metabolomics library. For LC–MS-based metabolomics analyses, feature identification was performed by comparing MS/MS spectra acquired at multiple collision energies (10, 20, and 40 eV) from QC samples with open-access MS/MS spectral libraries (positive and negative ion modes, RIKEN) integrated within MS-DIAL. Putative metabolite identifications were performed using accurate mass measurements, isotopic pattern analysis, and MS/MS spectral similarity (Metabolomics Standards Initiative, level 2). Data matrices obtained from GC–MS and LC–MS analyses were normalized against the total peak area of the corresponding total ion chromatograms. QC samples were used to monitor signal drift and assess analytical variability throughout the analytical batches. Analytical batch effects were further evaluated by visual inspection of QC clustering in unsupervised PCA. Tight clustering of QC samples indicated stable analytical performance, and therefore no additional batch-correction algorithm was applied. Metabolite features were excluded from further analysis if they exhibited more than 70% missing values, a relative standard deviation (RSD; batch-to-batch standard deviation, BSS) greater than 30% in QC samples, or were detected in blank or mobile phase controls. Remaining missing values were imputed using one-fifth of the minimum detected value for each metabolite. Fold changes were calculated using TIC-normalized relative metabolite intensities derived from the untargeted datasets. Therefore, reported fold change values represent relative differences in normalized signal intensity across time points rather than absolute concentration changes.

Following data preprocessing, GC–MS and LC–MS datasets were merged and analyzed using MetaboAnalyst 6.0. Principal component analysis (PCA) was first performed as an unsupervised exploratory method to assess overall data structure, evaluate analytical reproducibility, identify potential outliers, and examine systematic variation across time points.

Outlier detection was performed using Hotelling’s T^2^ statistic at the 95% confidence level based on PCA score plots generated in MetaboAnalyst. Samples located outside the Hotelling’s T^2^ confidence ellipse were considered statistical outliers and were removed from the dataset prior to all downstream univariate, multivariate, and longitudinal statistical analyses.

Subsequently, longitudinal comparisons were conducted using baseline (T0) as the reference (T0–T1, T0–T2, and T0–T3) to evaluate time-dependent metabolic changes during TPN administration. Time was treated as a continuous variable in the limma framework to assess monotonic temporal trends across T0, T1, T2, and T3, while baseline-referenced contrasts (T0–T1, T0–T2, and T0–T3) were used to identify time-point–specific changes.

Linear models were fitted within the limma framework, as implemented in MetaboAnalyst (Time Series Only analysis option), with time as the primary variable and T0 as the reference level. The subject identifier was included as a fixed-effect covariate in the model (metabolite ~ time + subject), thereby controlling for within-subject variability across sampling points.

A pathway analysis was performed using the Pathway Analysis module in MetaboAnalyst, incorporating all nominally significant metabolites (raw *p* < 0.05) identified at each time point. Enrichment analysis was conducted using the hypergeometric test, and pathway topology analysis was performed using relative betweenness centrality to calculate pathway impact scores.

## 3. Results

### 3.1. Baseline Characteristics and TPN Exposure

Baseline demographic and clinical characteristics of the study population are summarized in Table 1. Baseline clinical covariates of the cohort are summarized in Supplementary Table S1.

During the first 72 h of TPN administration (corresponding to T1–T3 sampling time points), delivered macronutrient amounts were summarized as grams per kilogram per day (g/kg/day). Across patient-days (n = 111), median (IQR) delivery was 0.60 (0.50–0.67) g/kg/day for amino acids, 1.74 (1.60–2.05) g/kg/day for dextrose, and 0.44 (0.39–0.57) g/kg/day for lipids. Ranges were 0.42–1.20, 1.19–3.39, and 0.27–1.32 g/kg/day, respectively. These ranges indicate moderate inter-individual variability in macronutrient composition and provide important context for interpreting the metabolomic results.

A total of 37 patients had paired plasma samples available at baseline (T0) and 24 h (T1). Among these, 33 patients had complete samples at T0, T1, and T2, and 28 patients had complete samples at T0, T1, T2, and T3. Missing samples at later time points were primarily due to hospital discharge or discontinuation of TPN therapy prior to the scheduled sampling time points. These numbers represent the clinical availability of samples at each time point prior to statistical outlier exclusion. Following Hotelling’s T^2^–based outlier removal, final sample numbers used in multivariate and longitudinal analyses are detailed in Section 3.2. A detailed overview of patient inclusion and sample availability across time points is provided in Supplementary Figure S1.

### 3.2. Global Metabolic Overview Revealed by Unsupervised PCA

**Figure 1:**
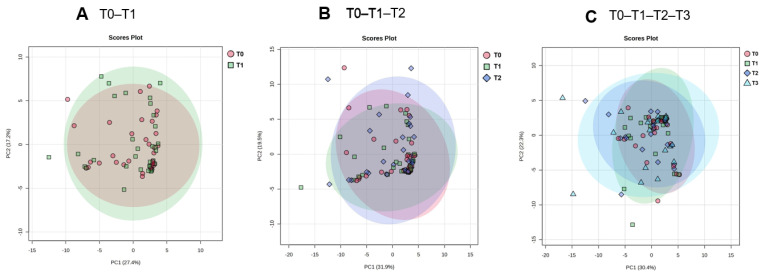
PCA score plots illustrating global metabolic variation in plasma samples collected during TPN. (**A**) PCA score plot based on samples collected at baseline (T0) and 24 h after TPN initiation (T1), including patients with complete data at both time points (n = 33). (**B**) PCA score plot based on samples collected at baseline (T0), 24 h (T1), and 48 h (T2), including patients with complete longitudinal data across all three time points (n = 31). (**C**) PCA score plot based on samples collected at baseline (T0), 24 h (T1), 48 h (T2), and 72 h (T3), including patients with complete longitudinal data across all four time points (n = 24). Outliers were identified using Hotelling’s T^2^ statistic at the 95% confidence level and excluded prior to downstream analyses. PCA score plots of the complete dataset before outlier exclusion are provided in Supplementary Figure S2.

PCA was applied to the combined GC–MS and LC–MS metabolomics dataset (Supplementary File S2) to examine global metabolic variation and time-dependent trends during the early postoperative period (Figure 1). PCA was conducted on sample subsets with complete longitudinal data for each comparison (T0–T1, n = 33; T0–T1–T2, n = 31; T0–T1–T2–T3, n = 24). Samples identified as statistical outliers based on Hotelling’s T^2^ (95% confidence ellipse) were excluded from further analyses (four samples in the T0–T1 comparison, two in T1–T2, and four in the T0–T1–T2–T3 comparison). The PCA score plots demonstrated partial, time-dependent clustering of samples collected before TPN initiation and at 24, 48, and 72 h postoperatively, with a gradual shift along the principal components indicating progressive metabolic remodeling. The observed overlap between time points likely reflects inter-individual metabolic heterogeneity and the individualized nature of postoperative parenteral nutrition. Importantly, time-dependent metabolic changes were more clearly captured by differential metabolite and longitudinal statistical analyses than by global PCA clustering alone. PCA was therefore used as an exploratory tool to visualize overall data structure and general temporal tendencies, whereas pathway-level and metabolite-level analyses provided greater sensitivity for detecting time-resolved metabolic alterations.

### 3.3. Early Time-Dependent Metabolic Changes Following TPN Initiation

To investigate metabolic changes associated with time during TPN administration, a linear model–based trend analysis was performed, treating time as a continuous variable across all sampling points (T0, T1, T2, and T3). Metabolites were ranked according to their correlation coefficients with time, and the top 25 metabolites showing the strongest positive and negative associations were identified for exploratory assessment.

**Figure 2:**
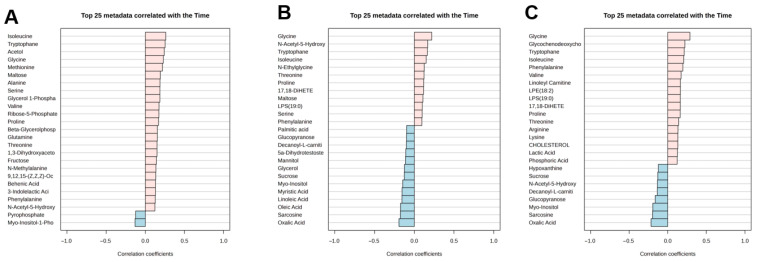
Top metabolites showing the strongest positive and negative associations with time during TPN administration based on linear model–based trend analysis (limma framework). Panels (**A**), (**B**), and (**C**) correspond to T0–T1, T0–T1–T2, and T0–T1–T2–T3 analyses, respectively. Bars represent model-derived correlation coefficients reflecting the direction and magnitude of association between normalized metabolite abundance and time.

As shown in Figure 2, metabolites exhibiting the strongest time-associated trends differed across the analyzed time windows. In the T0–T1 comparison (Figure 2A), isoleucine, tryptophan, and acetol showed the strongest positive correlations with time. In the T0–T1–T2 analysis (Figure 2B), glycine, N-acetyl-5-hydroxytryptamine, and tryptophan emerged as the most strongly positively correlated metabolites. In the full T0–T1–T2–T3 analysis (Figure 2C), glycine, glycochenodeoxycholic acid, and tryptophan exhibited the strongest positive associations with time. Conversely, metabolites such as sarcosine, oxalic acid, and myo-inositol consistently demonstrated negative correlations across extended time points. To reduce the risk of overinterpretation, downstream interpretation focused on metabolites showing consistent time-associated trends across multiple analyses rather than isolated statistical significance. Correlation coefficients shown in Figure 2 were derived from the linear model–based time trend analysis (limma framework) and reflect the direction and magnitude of association between normalized metabolite abundance and time.

**Figure 3:**
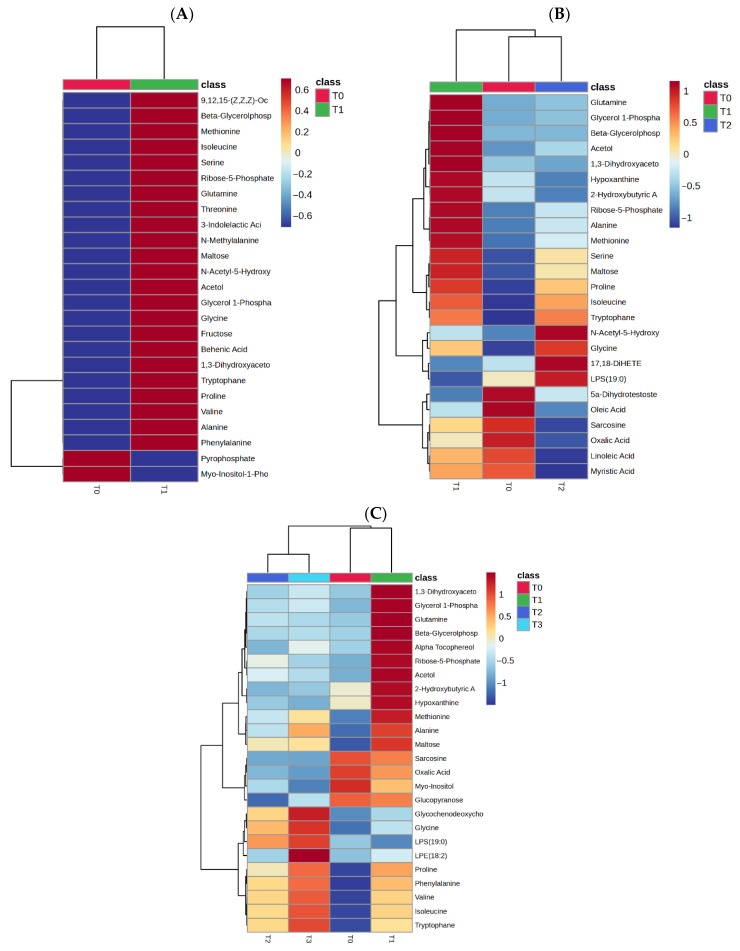
Heatmap visualization of time-dependent metabolite abundance patterns during total parenteral nutrition (TPN). Heatmaps were generated using mean metabolite intensities at each time point: (**A**) baseline (T0) and 24 h after TPN initiation (T1), (**B**) baseline (T0), 24 h (T1), and 48 h (T2), and (**C**) baseline (T0), 24 h (T1), 48 h (T2), and 72 h (T3). Rows represent metabolites showing nominally significant time-dependent changes or consistent temporal trends, and columns represent time points. Values are displayed as z-score-normalized mean relative abundances. Metabolites were clustered using hierarchical clustering with Euclidean distance and Ward’s linkage. Color intensity indicates relative increases or decreases in metabolite levels across time, illustrating progressive increases in several amino acids together with decreases in selected small organic acids during early TPN support.

To further illustrate the temporal behavior of selected metabolites, heatmap-based visualizations were generated to summarize relative changes in metabolite abundances across time points (Figure 3). Metabolites included in the heatmaps were selected from those showing nominally significant time-dependent changes in the corresponding comparisons or consistent trends in the linear model-based correlation analysis. Metabolites were clustered using hierarchical clustering with Euclidean distance and Ward’s linkage. Overall, the heatmaps demonstrate gradual and coordinated temporal changes, characterized mainly by increasing levels of several amino acids, including glycine, isoleucine, valine, phenylalanine, alanine, and tryptophan, together with decreasing levels of sarcosine, oxalic acid, myo-inositol, and glucopyranose, consistent with progressive metabolic adaptation during early TPN support.

Overall, these findings indicate that TPN administration is accompanied by systematic and time-dependent alterations in plasma metabolite profiles during the first 72 h following gastrointestinal surgery, particularly involving amino acid-related metabolic processes.

### 3.4. Time-Point–Specific Metabolic Changes Relative to Baseline (T0)

To further characterize time-dependent metabolic alterations during TPN, pairwise comparisons were performed between each postoperative time point and baseline (T0). Differential metabolite analysis was conducted for T1 vs. T0, T2 vs. T0, and T3 vs. T0 using a linear model–based contrast approach, allowing identification of metabolites exhibiting nominally significant deviations from baseline at each time point. To address multiple testing considerations, raw *p*-values were evaluated, and metabolites with *p* < 0.05 were considered nominally significant. Given the exploratory design of this longitudinal metabolomics study and the relatively limited sample size, the primary objective was to identify time-dependent metabolic trends rather than to establish definitive biomarkers. Accordingly, findings are presented as hypothesis-generating and should be interpreted in this context.

#### 3.4.1. T1 vs. T0 (24 h)

At 24 h after TPN initiation, early metabolic changes were primarily observed in amino acid–related pathways. In the T0–T1 comparison, fold changes (FC) were 1.58 for tryptophan (*p* = 0.0029), 1.38 for isoleucine (*p* = 0.0206), 2.01 for methionine (*p* = 0.0329), and 1.25 for glycine (*p* = 0.0315), indicating an early increase in circulating amino acid levels. In contrast, selected small organic acids demonstrated relative decreases, including sarcosine (FC = 0.88) and oxalic acid (FC = 0.81), suggesting an initial reorganization of one-carbon–related and carbohydrate-associated metabolic pathways during the immediate postoperative phase.

From a lipid-related perspective, decanoyl-L-carnitine also decreased at T1 relative to baseline (FC = 0.61, *p* = 0.0330).

#### 3.4.2. T2 vs. T0 (48 h)

At 48 h after TPN initiation, these trends became more pronounced and extended to a broader set of metabolites. The amino acid–related response expanded to include glycine, isoleucine, valine, phenylalanine, proline, alanine, and tryptophan.

In the T0–T2 comparison, glycine (FC = 1.35, *p* = 0.0100) and tryptophan (FC = 1.47, *p* = 0.0472) continued to increase relative to baseline. Reductions in sarcosine (FC = 0.54), oxalic acid (FC = 0.51), and myo-inositol (FC = 0.73) persisted, indicating progressive metabolic adaptation rather than transient fluctuations.

The decrease in decanoyl-L-carnitine also persisted at this time point (FC = 0.64, *p* = 0.0312).

#### 3.4.3. T3 vs. T0 (72 h)

By 72 h following TPN initiation, the magnitude and consistency of metabolic differences relative to baseline were further accentuated. In the T0–T3 comparison, amino acids involved in protein and nitrogen metabolism exhibited more pronounced increases, including glycine (FC = 1.51, *p* = 0.0011), isoleucine (FC = 1.66, *p* = 0.0059), valine (FC = 1.36, *p* = 0.0317), phenylalanine (FC = 1.58, *p* = 0.0177), proline (FC = 1.49, *p* = 0.0425), and tryptophan (FC = 1.98, *p* = 0.0061).

In parallel, carbohydrate- and energy-related metabolites displayed nominally significant time-dependent alterations. The T0–T3 comparison revealed increases in acetol (FC = 1.12, *p* = 0.0071), maltose (FC = 2.34, *p* = 0.0199), glycerol-1-phosphate (FC = 1.20, *p* = 0.0299), and fructose (FC = 1.35, *p* = 0.0421), whereas glucopyranose showed a relative decrease (FC = 0.78, *p* = 0.0216). Lactate levels also increased at 72 h (FC = 1.67, *p* = 0.0468).

Additionally, lipid-related alterations became evident at 72 h, with nominally significant increases detected in long-chain acylcarnitines and lysophospholipids. Linoleyl-carnitine increased (FC = 2.92, *p* = 0.0260), LPE(18:2) increased (FC = 2.64, *p* = 0.0460), and LPC(18:0) increased (FC = 1.68, *p* = 0.0460).

**Figure 4:**
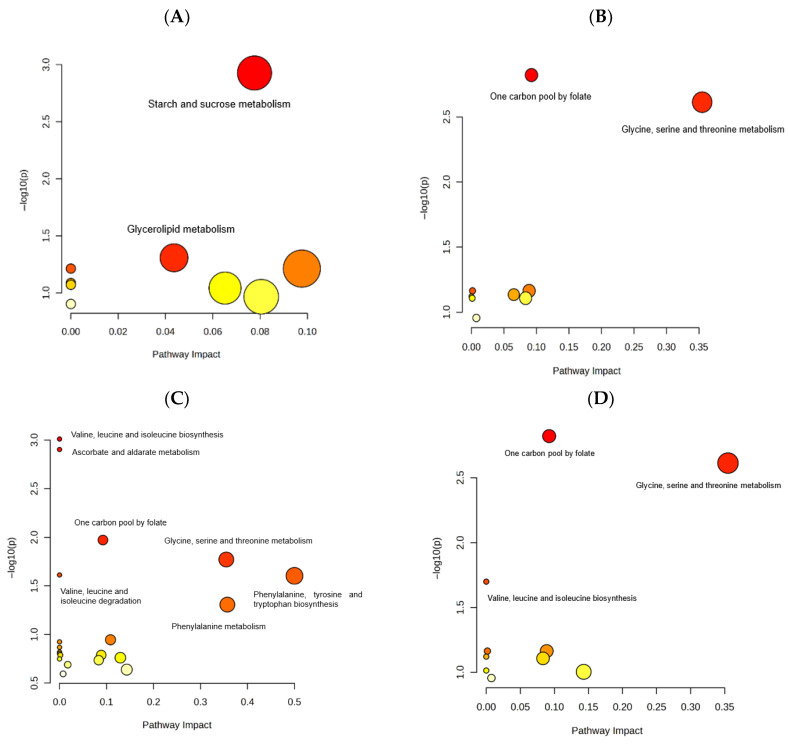
Time-resolved visualization of pathway-level metabolic changes during TPN based on longitudinal data. Panels (**A**), (**B**), (**C**), and (**D**) correspond to baseline-referenced comparisons of T0–T1, T0–T2, and T0–T3 within the full T0–T1–T2–T3 dataset, and a simultaneous analysis across all time points (T0–T1–T2–T3), respectively.

Time-resolved pathway impact analysis summarizing baseline-referenced metabolic alterations across time points is presented in Figure 4, with detailed pathway statistics provided in Supplementary File S2. Together, these time-point-specific comparisons demonstrate that TPN administration is associated with a gradual and structured metabolic transition, characterized by early and sustained deviations from baseline rather than isolated or transient effects.

### 3.5. Descriptive Pathway-Level Overview of Time-Dependent Metabolic Alterations

To place the observed time-dependent metabolite changes into a broader biological context, a descriptive pathway-level overview was generated based on metabolites exhibiting consistent deviations from baseline across multiple time points (Figure 4). Rather than performing a formal pathway enrichment analysis, metabolites identified in the time-point-specific comparisons were mapped to their associated metabolic pathways using curated biochemical knowledge, with the aim of providing an integrative and exploratory interpretation of the observed trends.

Metabolites showing sustained increases over time were predominantly associated with amino acid-related pathways, including glycine, serine and threonine metabolism, branched-chain amino acid metabolism, and phenylalanine and tryptophan metabolism. In addition, early pathway-level signals involved carbohydrate- and lipid-related processes, including starch and sucrose metabolism and glycerolipid metabolism, particularly at the earliest time point comparisons. These pathway-level associations are consistent with coordinated changes in amino acid availability and nitrogen handling during the early course of total parenteral nutrition. Conversely, metabolites demonstrating sustained decreases were linked to pathways related to one-carbon metabolism, inositol phosphate metabolism, and broader carbon metabolism. These pathway-level associations are consistent with time-dependent alterations in carbon flux and small-molecule metabolism observed following TPN initiation.

Overall, this pathway-level overview complements the metabolite-level and time-point-specific analyses by providing a systems-oriented interpretation of the metabolic adaptations occurring during the early postoperative TPN period.

## 4. Discussion

The aim of this study was to characterize early, time-dependent systemic metabolic adaptations during the first 72 h of total parenteral nutrition following gastrointestinal surgery using a longitudinal untargeted metabolomics approach. By combining complementary GC–MS and LC–MS platforms with time-resolved statistical analyses across multiple postoperative sampling points, this study enabled evaluation of global metabolic trajectories. It also allowed assessment of pathway-specific metabolic changes during early TPN support. The metabolic alterations observed in this study represent early metabolic signatures during the initial phase of TPN administration rather than clinical endpoints and should be interpreted in the context of short-term postoperative metabolic adaptation. Interpretation of pathway-related changes in this study is based on relative differences in metabolite abundances observed across time points. Although fold change values are derived from TIC-normalized signal intensities in an untargeted metabolomics framework, they represent relative differences in normalized metabolite abundance across time points rather than absolute concentration changes. In longitudinal metabolomic studies, coordinated and statistically supported changes across multiple metabolites and time points may indicate biologically relevant metabolic adjustments, even when individual effect sizes are modest. Accordingly, the present findings are interpreted as consistent temporal trends in systemic metabolism rather than large-amplitude concentration changes.

Plasma metabolomic profiles were compared in patients receiving TPN following gastrointestinal surgery at baseline prior to TPN initiation (T0) and at 24, 48, and 72 h thereafter (T1–T3). The early postoperative period is classically characterized by a stress-related metabolic phenotype, including increased proteolysis, enhanced gluconeogenesis, altered fatty acid oxidation, and negative nitrogen balance [18,19]. As emphasized in perioperative nutrition studies and ESPEN surgical nutrition guidelines, early TPN aims to attenuate this catabolic response by providing adequate energy and substrates and facilitating the transition toward metabolic stabilization [20,21].

Overall, the metabolomic data in the present study indicate a distinct early metabolic remodeling characterized by pronounced alterations in amino acid metabolism, reorganization of carbohydrate-related and glycolytic pathways, and specific changes in lipid–carnitine–bile acid metabolism. Similar patterns have been reported in metabolomic studies of early postoperative TPN, including pancreatic surgery cohorts, as well as in experimental models of parenteral nutrition [22,23].

Since baseline (T0) samples were collected in the early postoperative period prior to initiation of TPN, the longitudinal metabolic changes observed in this study likely reflect the combined influence of postoperative recovery processes and parenteral nutritional support. Therefore, the present findings are interpreted as associative and descriptive of early postoperative metabolic adaptation rather than as direct causal effects of TPN alone.

### 4.1. Amino Acid Metabolism

Following TPN initiation, plasma amino acid concentrations increased markedly, particularly between 24 and 72 h. Within the first 24 h, increases were observed in tryptophan, isoleucine, methionine, and glycine. These early changes were followed by a progressive expansion of the amino acid response at later time points, involving multiple essential, branched-chain, and glucogenic amino acids. These findings are consistent with previous TPN and metabolomics studies reporting gradual replenishment of circulating amino acid pools during early nutritional support [23,24].

The postoperative stress response is typically associated with increased proteolysis, skeletal muscle breakdown, and negative nitrogen balance. Under these conditions, hepatic gluconeogenesis relies heavily on amino acids released from muscle tissue—particularly alanine and glutamine—which are converted to glucose in the liver. Branched-chain amino acids (BCAAs; leucine, isoleucine, valine) are transaminated in skeletal muscle to form alanine and glutamine, thereby supporting systemic gluconeogenesis [25]. Post-TPN increases in circulating BCAAs, particularly isoleucine and valine, may be associated with partial attenuation of surgery-associated proteolysis. These changes may also reflect early preservation of muscle protein pools. Randomized and observational studies in surgical and critically ill patients have reported that BCAA-enriched parenteral formulations may stimulate protein synthesis, reduce proteolysis, and partially improve nitrogen balance and muscle-related outcomes [26,27]. In addition, increases in aromatic amino acids (phenylalanine and tryptophan) and glucogenic amino acids (alanine and proline) likely reflect both the amino acid load provided by TPN solutions and expansion of the circulating free amino acid pool as proteolysis decreases and nitrogen balance improves. Similar temporal patterns have been reported in metabolomic and targeted amino acid studies following postoperative TPN, with amino acid levels and urea cycle intermediates gradually approaching values observed in healthy controls [23,28].

Glycine emerged as one of the most responsive metabolites, showing increased levels at both early (24 h) and later (48–72 h) time points. Glycine is a precursor for glutathione (GSH) synthesis, a major constituent of collagen and connective tissue proteins, and a key component of one-carbon (C1) metabolism involved in nucleotide synthesis and methylation reactions [29]. Several studies have suggested that glycine availability may be rate-limiting for GSH synthesis, particularly under conditions of oxidative stress, and that glycine supplementation can enhance tissue GSH levels and antioxidant defense [30]. Accordingly, increases in glycine and alanine may be associated with reorganization of energy metabolism. They may also indicate early restoration of substrates required for hepatic protein synthesis, collagen deposition, and antioxidant defense during postoperative recovery. When interpreted alongside clinical recovery, expansion of the glycine–alanine pool may support wound healing, immune function, and tissue repair.

Alanine is classically linked to the glucose–alanine cycle and gluconeogenesis, supporting hepatic glucose production during stress [25]. When adequate carbohydrate and amino acid supply is provided by TPN, reliance on gluconeogenesis may decrease and alanine may be redirected toward protein synthesis and other biosynthetic pathways; therefore, the alanine increase may reflect improved substrate availability for anabolic processes.

### 4.2. One-Carbon Metabolism

At later postoperative time points, metabolites associated with one-carbon metabolism, including sarcosine and oxalic acid, showed a decreasing trend. Sarcosine is an intermediate in glycine–serine and one-carbon metabolism, generated via S-adenosylmethionine (SAM)-dependent methylation of glycine and convertible back to glycine. This cycle regulates the cellular SAM/S-adenosylhomocysteine (SAH) ratio and thereby influences methylation potential, DNA and histone methylation, and nucleotide synthesis. Recent studies have highlighted the importance of serine–glycine and one-carbon metabolism in nucleotide biosynthesis, redox homeostasis, and epigenetic regulation, particularly under stress or increased proliferative demand [31,32]. In many proliferative systems, serine acts as the dominant one-carbon donor, while glycine is preferentially consumed for GSH and structural protein synthesis [33].

In the present study, increasing glycine levels alongside decreasing sarcosine levels may indicate preferential utilization of glycine for antioxidant defense and collagen synthesis. This pattern may also reflect reorganization of the one-carbon metabolic pool during early postoperative recovery. The reduction in oxalic acid, a metabolite linked to ascorbate and glyoxylate metabolism, may reflect a decrease in oxidative stress–associated organic acid burden; however, given the limited data on this pathway, this interpretation should be considered exploratory.

Overall, the combined pattern of increased glycine and decreased sarcosine and oxalic acid is consistent with previous metabolomic studies reporting reorganization of one-carbon metabolism during early postoperative nutritional support.

### 4.3. Carbohydrate Metabolism

Characteristic changes in carbohydrate-related metabolites were observed during the early phase, with increases in maltose, fructose, glycerol-1-phosphate, and acetol within the first 24–48 h. These alterations became more evident at later time points, accompanied by a relative decrease in glucopyranose and an increase in lactate levels, indicating enhanced glycolytic activity during early TPN support. Collectively, these findings are consistent with increased utilization of glycolysis-related pathways and lactate production during the early postoperative phase. Similar increases in glycolysis-related intermediates and lactate have been reported in surgical and critically ill patients receiving parenteral or mixed nutritional support [23,34].

Surgical trauma and critical illness are commonly associated with insulin resistance, increased hepatic gluconeogenesis, elevated endogenous glucose production, and hyperglycemia [18,19]. Under these conditions, glucose and glycolysis become primary energy sources, while fatty acid oxidation is often impaired. Exogenous glucose provided by TPN can reduce dependence on gluconeogenic substrates but may also promote hyperglycemia and lactate accumulation. Reviews on lactate metabolism in critical illness emphasize that elevated lactate may arise not only from tissue hypoxia/hypoperfusion but also from catecholamine-driven glycolysis, Na^+^/K^+^-ATPase activation, and inflammatory signaling, reflecting accelerated glycolytic flux [35,36,37].

Although only a limited number of TCA cycle intermediates reached statistical significance, the observed increase in lactate and the changes in sugar-related metabolites are consistent with rapid activation of glucose-based energy production during early TPN [23,38]. The relative decrease in glucopyranose signals may reflect enhanced tissue glucose uptake under insulin influence and improved hemodynamic status. The modest lactate increase observed at 72 h is more likely indicative of high glycolytic flux and a transient imbalance between glycolysis and mitochondrial oxidation. It is unlikely to reflect overt hypoperfusion [37]. Overall, these findings are consistent with rapid changes in carbohydrate-based energy metabolism, contributing to restoration of metabolic balance by reducing reliance on proteolysis and fatty acid oxidation [18,39].

Considering that a substantial proportion of patients undergoing major gastrointestinal surgery may have underlying malignancies, the broader implications of TPN-associated metabolic modulation in oncologic contexts warrant further investigation in appropriately designed studies.

### 4.4. Lipid, Carnitine, and Bile Acid Metabolism

Time-dependent alterations in lipid-related metabolites were observed during early TPN administration, involving acylcarnitines, lysophospholipids, and bile acid derivatives. Early reductions in medium-chain acylcarnitines were followed by increases in long-chain acylcarnitines and lysophospholipids at later time points, suggesting dynamic remodeling of fatty acid oxidation and phospholipid metabolism. Taken together, these findings are consistent with dynamic postoperative changes in metabolites related to fatty acid oxidation and phospholipid metabolism during TPN administration. Similar alterations in acylcarnitine and lysophospholipid profiles have been reported in metabolomic studies of parenteral lipid emulsions and critical illness [22,23].

Acylcarnitines are intermediates of fatty acid β-oxidation and are essential for mitochondrial transport of long-chain fatty acids. Alterations in acylcarnitine profiles have been shown to reflect mitochondrial function, β-oxidation efficiency, and disease severity in critical illness and cardiometabolic disorders [40,41,42]. Accumulation of short- and medium-chain acylcarnitines is often interpreted as a marker of incomplete β-oxidation and mitochondrial substrate overload [43]. In this context, the early decrease in decanoyl-L-carnitine (24–48 h) may indicate a transition from intense stress-induced lipolysis and fatty acid mobilization toward more controlled and efficient fatty acid utilization. By providing adequate carbohydrate and amino acid substrates, TPN may reduce reliance on fatty acid oxidation as the sole energy source, thereby limiting accumulation of intermediate acylcarnitines. Conversely, the later increase in long-chain acylcarnitines and lysophospholipids at 72 h may reflect uptake and processing of TPN-derived lipid emulsions by hepatic and peripheral tissues, together with activation of phospholipid turnover. The later increase in long-chain acylcarnitines at 72 h is consistent with previous reports describing altered acylcarnitine profiles during early parenteral nutrition and critical illness [22,23,40,41,42,43]. In such settings, elevated acylcarnitine species may indicate partial bottlenecks in mitochondrial fatty acid flux rather than efficient energy production. This pattern may be associated with early TPN lipid delivery combined with transient postoperative mitochondrial stress. Moreover, acylcarnitines are increasingly recognized as bioactive lipid intermediates capable of modulating inflammatory signaling pathways [43]. Their elevation may therefore reflect not only altered substrate handling but also integration of metabolic and inflammatory responses during early recovery.

Similarly, the concurrent rise in lysophospholipid species may represent active membrane remodeling rather than passive incorporation of exogenous lipids. Lysophospholipids participate in membrane turnover, lipoprotein remodeling, and inflammatory signaling cascades. In the postoperative context, increased membrane repair, immune activation, and hepatocellular adaptation to parenteral nutrient exposure may collectively contribute to this pattern [22,44,45,46]. Taken together, the lipid–carnitine alterations observed at 72 h likely reflect a complex interplay between substrate supply, mitochondrial oxidative capacity, and adaptive membrane remodeling. They are unlikely to represent a single isolated mechanism.

Animal models and postoperative TPN studies have reported increased phospholipid and long-chain fatty acid metabolism as part of an anabolic response associated with tissue repair [22,23]. Accordingly, the early metabolic signals observed here may represent precursors of longer-term adaptive lipid metabolic remodeling.

Lysophosphatidylcholine (LPC) and lysophosphatidylethanolamine (LPE) are intermediates in membrane phospholipid turnover and play roles in membrane remodeling, lipoprotein metabolism, inflammation, and immune modulation. In TPN-treated surgical patients, reorganization of phospholipid metabolism is important for tissue repair, membrane integrity, and lipoprotein transport [22]. The observed increases in LPC(18:0) and LPE(18:2) suggest incorporation of TPN-derived lipids into phospholipid pools and activation of membrane phospholipid cycling. Furthermore, the increase in the conjugated bile acid glycochenodeoxycholic acid 3-glucuronide suggests an early hepatic response of bile acid synthesis and conjugation to parenteral nutrient load. Experimental and clinical studies have demonstrated that parenteral nutrition and lack of enteral stimulation can affect bile acid synthesis, pool size, and enterohepatic circulation, contributing to the pathogenesis of parenteral nutrition-associated cholestasis [44,45,46]. Although the present study is limited to the first 72 h and does not allow conclusions regarding long-term hepatic outcomes, the observed increase in this conjugated bile acid may represent an early metabolic signal of hepatic adaptation to parenteral nutrition.

### 4.5. Overall Interpretation and Clinical Implications

A key strength of this study is its longitudinal design, which enabled characterization of dynamic metabolic trajectories rather than static differences at isolated time points. Several metabolic changes—particularly in amino acid pools, glycolysis-related intermediates, and lipid–carnitine profiles—evolved progressively over time rather than occurring uniformly across all comparisons, underscoring the importance of time-resolved metabolomic assessment during the early postoperative period.

In this context, the present study provides a complementary perspective to existing literature by focusing on repeated plasma sampling within the same gastrointestinal surgery patients from the immediate pre-TPN postoperative baseline through the first 72 h of TPN administration. The longitudinal design, together with the combined use of GC–MS and LC–MS platforms, enables broader metabolite coverage and supports a time-resolved description of early systemic metabolic signatures during the initial phase of parenteral nutrition. This approach extends previous work that has predominantly relied on cross-sectional comparisons or later postoperative time points.

Collectively, these metabolomic analyses provide a multidimensional view of the metabolic transition occurring during the first 72 h of TPN in patients undergoing gastrointestinal surgery. The findings are largely consistent with previous metabolomic and biochemical studies of TPN and critical illness and support the concept that early TPN is associated with a transition from a stress-dominated catabolic state toward a more regulated metabolic profile [24,25,29]. From a clinical perspective, these results suggest that early TPN may support postoperative recovery by meeting macronutrient requirements. It may also facilitate coordinated metabolic adaptation at the pathway level. Time-dependent restoration of amino acid availability, changes in glucose-based energy production, and reorganization of lipid and phospholipid metabolism may collectively contribute to improved metabolic efficiency during the critical early postoperative phase. These findings provide an overview of early metabolic adaptations to TPN and do not imply direct clinical outcomes.

Although direct associations between metabolomic alterations and specific clinical outcomes (such as length of hospital stay, return of bowel function, or postoperative infections) were not examined in this study, the identified early metabolic signatures may still hold clinical relevance. The observed time-dependent changes in amino acid pools, carbohydrate-related metabolites, and lipid–carnitine profiles likely reflect systemic metabolic adaptation to early parenteral nutrition and may serve as a basis for future investigations linking metabolite trajectories with postoperative recovery or complication risk. In this context, combining metabolomic data with routinely collected clinical laboratory parameters (e.g., CRP, albumin, or inflammatory markers) in larger, well-characterized cohorts could help to better define the translational significance of these findings.

### 4.6. Limitations

This study has several limitations that should be acknowledged. First, metabolomic analyses were limited to the early postoperative period, precluding assessment of longer-term metabolic adaptations during TPN support. Second, TPN formulations were individualized according to clinical needs rather than standardized, which may have contributed to inter-individual variability. This variability, however, was not expected to substantially limit the metabolomic analyses, as the study was designed to capture systemic metabolic responses to parenteral nutrition under real-world clinical conditions rather than to compare the effects of specific nutrient formulations. Third, the untargeted metabolomics approach provides relative changes in metabolite levels and does not allow direct inference of absolute metabolic fluxes or tissue-specific activity. Finally, the observational design limits causal interpretation of the associations observed between TPN exposure and metabolic profiles. Future studies integrating extended longitudinal follow-up, targeted metabolomic validation, and linkage to clinical outcomes may further clarify the clinical and translational relevance of these early metabolic trajectories.

In addition, key metabolites identified in the untargeted analyses were not independently validated using targeted quantitative assays, and future studies incorporating targeted metabolomics will be important to confirm these findings. Furthermore, the absence of a parallel control group (e.g., patients receiving enteral nutrition or no nutritional support) further limits causal interpretation of TPN-specific effects.

A reduction in sample numbers across longitudinal subsets was primarily attributable to hospital discharge or discontinuation of TPN therapy prior to scheduled sampling time points. Consequently, not all patients contributed samples at later time points. Samples identified as statistical outliers based on Hotelling’s T^2^ (95% confidence ellipse) were excluded to prevent undue influence of extreme multivariate observations. Missingness was not associated with documented clinical deterioration. The statistical modeling approach used in this study allowed inclusion of participants even when some follow-up samples were missing, thereby partially mitigating the impact of incomplete longitudinal data. Nevertheless, future studies with larger cohorts and fully balanced longitudinal sampling will be valuable to further validate the robustness and generalizability of these time-dependent metabolic patterns.

## 5. Conclusions

In conclusion, this study demonstrates that initiation of total parenteral nutrition following gastrointestinal surgery is associated with distinct and time-dependent alterations in the plasma metabolome during the first 72 h. Early TPN was accompanied by progressive replenishment of circulating amino acid pools and activation of glucose-based energy metabolism, alongside coordinated changes in lipid and carnitine-related pathways, consistent with an early transition from a stress-related catabolic state toward a more regulated and energy-efficient metabolic profile. By providing a time-resolved, systems-level view of metabolic adaptation during early postoperative TPN, this work highlights the utility of untargeted metabolomics for characterizing complex metabolic responses to parenteral nutrition and supports its potential value for monitoring metabolic recovery in surgical patients.

## Figures and Tables

**Table 1:** Baseline demographic and clinical characteristics of the study population (n = 37).

Variable	Value
Age (years), mean ± SD	61.2 ± 17.0
Sex, n (%)	Male: 22 (59.5%) Female: 15 (40.5%)
BMI (kg/m^2^), mean ± SD	25.0 ± 3.2
Primary surgical procedure (index surgery), n (%)	
Colorectal surgery	17 (45.9%)
Gastric/esophageal surgery	9 (24.3%)
Small bowel surgery	5 (13.5%)
Pancreatic surgery	3 (8.1%)
Multivisceral/cytoreductive surgery	3 (8.1%)

BMI values were calculated from measured body weight and height.

## Data Availability

The original contributions presented in this study are included in the article/Supplementary Material. Further inquiries can be directed to the corresponding author.

## Supplementary Materials

The following supporting information can be downloaded at: https://www.mdpi.com/article/10.3390/metabo16030199/s1. Table S1. Baseline clinical covariates of the cohort (n = 37); Figure S1. Study flow diagram illustrating patient inclusion, longitudinal sample availability, and statistical outlier exclusion. Figure S2. PCA score plots of the complete metabolomics dataset before outlier exclusion.
