# Supplementary material for: Longitudinal Plasma Metabolomics by GC–MS and LC–MS During Total Parenteral Nutrition After Gastrointestinal Surgery
Source: Metabolites. 2026 Mar 16;16(3):199. doi: 10.3390/metabo16030199 (PMC13027661; doi:10.3390/metabo16030199)
Supplement: Supplementary file 1 [file metabolites-16-00199-s001.zip › metabolites-4156173-supplementary/Supplemantary.pdf]

Table S1. Baseline clinical covariates of the cohort (n = 37)

**Variable**	**Value**
Age (years), mean ± SD	61.2 ± 17.0
Male sex, n (%)	22 (56.4)
Female sex, n (%)	15 (43.6)
CRP (mg/L), median (IQR)	12 (4–30)
Creatinine (mg/dL), mean ± SD	0.94 ± 0.76
AST (U/L), median (IQR)	29 (20–46)
ALT (U/L), median (IQR)	16 (10–37)
GGT (U/L), median (IQR)	44 (18–66)
LDH (U/L), median (IQR)	185 (141–248)

Patients enrolled in the study (n = 37)
Adult patients undergoing major gastrointestinal surgery and receiving postoperative TPN.

↓

Available for baseline and 24 h sampling (T0–T1) (n = 37)

↓

Available for 48 h longitudinal comparison (T0–T1–T2) (n = 33)
Missing at T2 (n = 4)
Reason: Hospital discharge or discontinuation of TPN prior to scheduled sampling.

↓

Available for 72 h longitudinal comparison (T0–T1–T2–T3) (n = 28)
Missing at T3 (n = 9)
Reason: Hospital discharge or discontinuation of TPN prior to scheduled sampling.

↓

Statistical outlier exclusion (Hotelling's T^2, 95% confidence ellipse)
Outliers removed prior to multivariate and longitudinal analyses.

↓

Final datasets used for statistical analyses:

T0–T1: n = 33

T0–T1–T2: n = 31

T0–T1–T2–T3: n = 24

Figure S1. Study flow diagram illustrating patient inclusion, longitudinal sample availability, and statistical outlier exclusion.

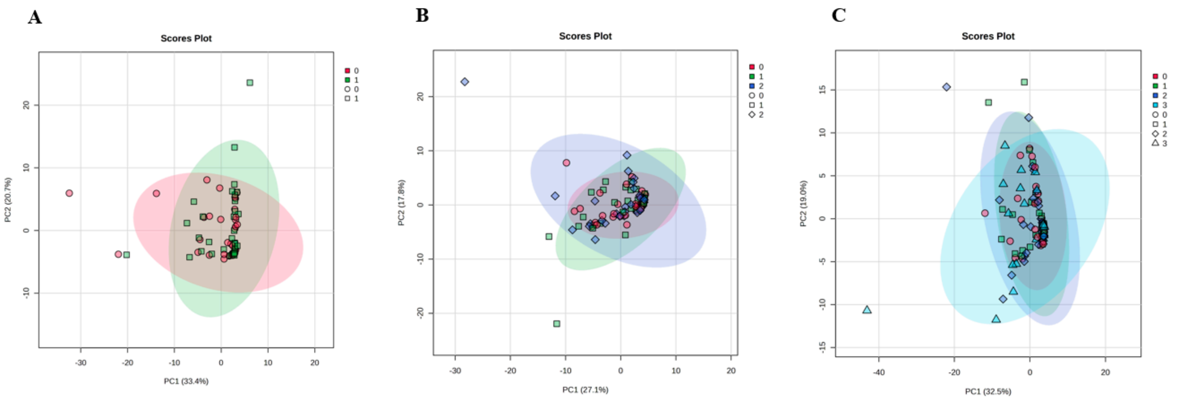

Figure S2. PCA score plots of the complete metabolomics dataset before outlier exclusion. (**A**) PCA score plot based on samples collected at baseline (T0) and 24 h after TPN initiation (T1), including patients with complete data at both time points (n = 37). (**B**) PCA score plot based on samples collected at baseline (T0), 24 h (T1), and 48 h (T2), including patients with complete longitudinal data across all three time points (n = 33). (**C**) PCA score plot based on samples collected at baseline (T0), 24 h (T1), 48 h (T2), and 72 h (T3), including patients with complete longitudinal data across all four time points (n = 28). Outliers were subsequently identified using Hotelling's T^2 statistic at the 95% confidence level and excluded prior to downstream analyses (see Figure 1).
